# Effects of propolis and gamma‐cyclodextrin on intestinal neoplasia in normal weight and obese mice

**DOI:** 10.1002/cam4.787

**Published:** 2016-06-05

**Authors:** Youngjin Cho, Linda Gutierrez, Michael Bordonaro, Daniel Russo, Frank Anzelmi, Jayde T. Hooven, Carmine Cerra, Darina L. Lazarova

**Affiliations:** ^1^ The Commonwealth Medical CollegeScrantonPennsylvania18509; ^2^ Wilkes UniversityWilkes‐BarrePennsylvania18766; ^3^ Penn State Hershey College of MedicineHersheyPennsylvania17033

**Keywords:** Colon cancer, gamma‐cyclodextrin, obesity, propolis, Western diet

## Abstract

Obesity is associated with colorectal cancer (CRC). This effect might be attributed to adipokine‐supported signaling. We have established that propolis suppresses survival signaling in CRC cells *in vitro*; therefore, we ascertained the ability of a propolis supplement to modulate intestinal neoplastic development in C57BL/6J‐*ApcMin/+*/J mice in the lean and obese state. To induce obesity, mice were fed with a Western diet containing 40% fat. Since the propolis supplement includes gamma‐cyclodextrin, the interventions included diets supplemented with or without gamma‐cyclodextrin. The animals were administered the following diets: (1) control diet, (2) control diet/gamma‐cyclodextrin, (3) control diet/propolis, (4) Western diet, (5) Western diet/gamma‐cyclodextrin, and (6) Western diet/propolis. Western diet, resulting in obesity, accelerated neoplastic progression, as evidenced by the larger size and higher grade dysplasia of the neoplasms. In the context of normal weight, gamma‐cyclodextrin and propolis affected neoplastic progression, as determined by the size of the lesions and their grade of dysplasia. A statistically significant decrease in the number of adenomas was detected in mice fed a control diet with the propolis supplement (61.8 ± 10.6 vs. 35.3 ± 7.6, *P *=* *0.008). Although there was no significant difference in the polyp numbers between the six groups, the mice with the lowest number and size of adenomas were those fed a Western diet with gamma‐cyclodextrin. This unexpected outcome might be explained by the increased levels of apoptosis detected in the intestinal tissues of these obese mice. We posit that butyrate derived from the metabolism of gamma‐cyclodextrin may contribute to the decreased neoplastic burden in the context of obesity; however, future studies are required to address this possibility.

## Introduction

Although the overall incidence of colorectal cancer has been declining in the past 35 years, the incidence of the disease has increased among 20‐ to 34‐year olds and, by 2030, there could be up to a 90% increase in colon cancer (CC) incidence in this age group 1. This shift in age of incidence is due in part to the increasing obesity among children and adolescents 2. Although the mechanisms by which obesity predisposes to intestinal neoplasia are debated, one possibility is that obesity‐associated cytokines (adipokines) collaborate with cancer‐initiating mutations to support neoplastic development in the large intestine. Adipose tissue and macrophages infiltrating this tissue secrete high levels of cytokines, including TNFalpha, interleukins, and WNT5A that activate AKT, ERK, JNK, and JAK/STAT signaling 3. Recently, analyses of data from The Cancer Genome Atlas (TCGA) revealed that obese CC patients (with body mass index of 30 or greater) have a lower number of driver gene mutations than normal weight CC patients 4. One possible explanation for this observation is that neoplasm‐promoting levels of survival pathways in obese individuals are maintained by adipokines, rather than driver gene mutations.

Although obesity modulates the risk for at least 10 types of cancer 5, the link between obesity and CC risk might be modified by diet in a complex manner. Diet directly affects neoplastic development in the colon because the neoplasms are exposed to the luminal content, in which dietary agents and metabolites are present at high concentrations. For example, butyrate, a short‐chain fatty acid, is a fermentation product of dietary fiber in the colon, and its physiological concentrations in the large intestine can reach up to 20 mmol/L 6, 7, 8, 9. Exposure of CC cells with mutations in the WNT pathway to physiological levels of butyrate hyperactivates WNT/beta‐catenin‐dependent gene transcription, and subsequently, leads to apoptosis 10. These effects of butyrate are highly relevant since most CCs exhibit deregulated WNT/beta‐catenin transcription due to mutations in the *adenomatous polyposis coli* (*APC*) gene, and this type of mutations is frequently the first to take place in intestinal neoplastic development 11. This prevalent mutation profile may explain why human colon adenoma cells (i.e., LT‐97) derived from the earliest stages of neoplastic development also exhibit apoptosis in the presence of butyrate 12. Unlike neoplastic cells, normal colonocytes metabolize butyrate as a source of energy 13; these cells do not hyperactivate WNT signaling (as they do not have mutations in the pathway) and do not commit to apoptosis in the presence of butyrate.

The apoptotic effect of butyrate on CC cells can be further increased by propolis, a honeybee product 14. Propolis is a complex mixture of flavonoids, terpenes, and caffeic acid phenethyl ester, and all of these compounds could exert anticancer effects 15. We have ascertained that the combination of butyrate and propolis induces high levels of apoptosis in colon adenoma and carcinoma cells, but not in normal colonic cells 16. Since propolis suppresses AKT and JAK/STAT pathways 14, 16 that might be activated in obesity, we reasoned that propolis may counteract intestinal neoplastic development and this effect might be most discernible in the context of obesity. To test this hypothesis, we utilized the mouse model of intestinal cancer C57BL/6J‐*ApcMin/+*/J (JAX Strain 002020) that develops adenomas due to a truncating mutation in *Apc* 17, 18. Although *APC* mutations in humans are manifested by colorectal adenomas, *ApcMin*/+ mice develop predominantly small intestinal lesions 17, 19, 20, a phenomenon explained by the different number of stem cell divisions in the two tissue types in humans and mice 21. Two research groups have reported that when the *ApcMin*/+ mice were fed different formulations of a Western diet, the mice exhibit increased adenoma size without changes in tumor multiplicity or increase their neoplastic burden. Day and colleagues have observed that the high‐fat diet does not change the number of polyps; however, the ratio of large to small polyps is higher in mice fed a high‐fat diet 22. Utilizing a different dietary formulation, Baltgalvis and colleagues have reported that a Western diet increases the number of polyps in *ApcMin*/+ mice by 75% compared to AIN‐76A (control diet) ‐ fed mice 23.

The objective of our study was to ascertain whether the additional neoplastic burden in mice associated with diet‐induced obesity is suppressed by a propolis supplement. We utilized *Propolis with Cyclopower* (Manuka Health New Zealand), which we have previously characterized *in vitro* 14, 16. This propolis supplement is formulated with the oligosaccharide gamma‐cyclodextrin; therefore, the oligosaccharide was included as a control supplement in the intervention diets.

## Materials and Methods

### Animals

All mice were maintained on a 12‐hour light and dark cycle at the animal facility in The Commonwealth Medical College. The protocol for mouse work was approved by the Institutional Animal Care and Use Committee at The Commonwealth Medical College. Thirty‐two male C57BL/6J‐*ApcMin/+*/J mice (Jackson Laboratories, USA) at 5 weeks of age were randomly assigned to six intervention groups (*n* = 5–6). Three groups were fed a control diet (AIN‐93G) without any supplements, with 6% gamma‐cyclodextrin (Cavamax W8, Wacker Chemical Corporation), or with 8% propolis supplement containing gamma‐cyclodextrin (*Propolis with CycloPower Powder*, Manuka Health New Zealand, Ltd, New Zealand). The amount of gamma‐cyclodextrin (6%) in the second intervention diet was the same as the amount of gamma‐cyclodextrin in the propolis powder‐supplemented intervention diet. Three groups of mice were fed a Western diet without supplements, with 6% gamma‐cyclodextrin, or with 8% propolis supplement containing gamma‐cyclodextrin. The Western diet (cat # 100244, Dyets Inc, Bethlehem, PA) provides approximately 40% of total calories from fat. The diets were given at libitum for 8 weeks. Cages were equipped with mesh bottoms to prevent coprophagia. Body weight was recorded every other workday. Food consumption was assessed by weighing the food added to each cage, and by accounting for any spillage. At the end of the experiment, the mice were killed and tissues (intestines, liver, and epididymal fat) were collected for the analyses.

### Tissue analyses

Intestines (small intestines and colons) were dissected, fixed in Histochoice MB (Electron Microscopy Sciences, Hatfield, PA) for 24 h, and transferred to 70% ethanol. Representative tissues with or without lesions were frozen in liquid nitrogen. Intestinal neoplasms were counted by three researchers, and the average number was calculated based upon these independent counts. To estimate the size of the lesions, pictures of the lesions were taken with AmScope MA‐1000 camera on a Nikon SMZ645 dissecting microscope, images were obtained using Nikon ACT‐1 software, and size (surface area) of the lesions was established with Image J. A minimum of 70% of all lesions per intestine were processed to obtain average size. For histological studies, we utilized the technique “Swiss roll” to analyze the entire length of the intestines 24. Histopathological assessments were based upon hematoxylin and eosin‐stained slides by two blinded for the intervention pathologists, and according to the nomenclature for intestinal neoplasia in mice 20. Low‐ and high‐grade dysplasia were determined as recommended by the nomenclature for intestinal neoplasia in mouse models 20.

### Cell culture and chemicals

The human colorectal cancer cell line HCT‐116, the normal human fetal colonic cell line CCD841CoN, and the murine fibroblast cell line 3T3‐L1, were from the American Tissue Culture Collection. Cells were grown in alpha‐MEM medium with 10% fetal bovine serum. Caffeic acid phenethyl ester (CAPE)‐enhanced propolis (*Propolis with CycloPower*) was a gift from Manuka Health New Zealand Ltd. For *in vitro* studies, propolis was dissolved in dimethyl sulfoxide at 100 mg/mL; the stock solution was kept at −80°C.

### Western blot analyses

Western blot analyses were performed as reported 25. The following antibodies were used: a) from Cell Signaling: anti‐phospho‐p44/42 Erk1/2 (Thr202/Tyr204), anti‐phospho‐Stat3 (Tyr705), anti‐total Stat3, anti‐Erk1/2, anti‐cleaved Caspase 3 (#9661) that recognizes the large fragment (17/19kD) of activated Caspase 3 resulting from cleavage adjacent to Asp175; b) from Santa Cruz Biotechnology: anti‐Ser473‐phospho‐Akt, anti‐Akt, anti‐phospho‐cJUN, anti‐c‐Jun, anti‐Bax; and c) from Sigma: anti‐beta ACTIN. Antibodies were utilized at a dilution of 1:1,000, except the ant‐Actin antibody utilized at 1:5,000 dilution. Lysates from control and neoplastic intestinal tissues were prepared with RIPA lysis and extraction buffer (# 89900), Halt protease inhibitor (# 87786), and Halt Phosphatase inhibitor (# 78420) from ThermoScientific, USA. Tissues were homogenized in a Dounce tissue grinder with RIPA buffer containing protease inhibitors, phosphatase inhibitors, PMSF, and EDTA, lysed on ice for 40 min, and centrifuged at 4°C for 15 min. Western blotting analyses were performed with 30 *μ*g of total protein as estimated by Bradford assays. Detection of signal was carried out with horseradish peroxidase conjugated secondary antibodies and chemiluminescence kits (Santa Cruz Biotechnology). Densitometry was performed using an Alpha Innotech MultiImage II, correcting for local background and normalizing for differences in ACTIN values.

### Exome sequencing and analysis

Mouse exome sequencing was performed on genomic DNA from paraffin‐embedded small intestines. DNA extraction, library preparation, Agilent SureSelect mouse (51 Mb) capture; HiSeq2500 PE100‐125, and DNAnexus platform exome analysis (In/Del, SNV and CNV analysis) were performed by Otogenetics (Norcross, GA). All data were analyzed with BWA mapping software (to map low‐divergent sequences against a large reference genome) and the Genome Analysis Toolkit (SNP/Indel pipeline, a software for analysis of high‐throughput sequencing data, developed at the Broad Institute). Single‐nucleotide polymorphism (SNP) annotation was carried out against reference genome GRCm38/mm10. The database of somatic mutations was searched for mutations in genes associated with cancer (listed in COSMIC, a catalog of somatic mutations in cancer (http://cancer.sanger.ac.uk/census/).

### Statistics

Data were presented as mean ± standard deviation (SD). Unpaired Student's t‐test analysis was used to determine the significance of statistical differences in the number of mutations between mice on a control diet and mice on a Western type diet. Differences were considered significant at *P *<* *0.05. Neoplastic burden (size/number of neoplasms) was analyzed by one‐way analysis‐of‐variance (ANOVA), with Tukey's post‐test calculations to adjust for multiple comparisons with 95% confidence.

## Results

### Body weight and food consumption

**Figure 1:**
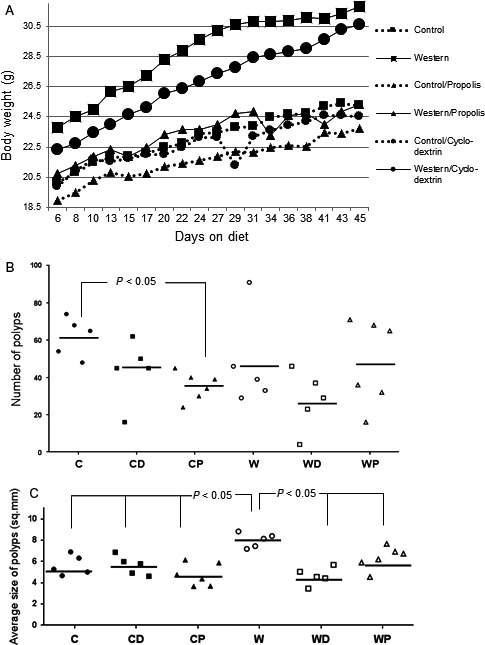
(A) The mouse body weight was measured every other workday for the entire duration of the study. (B) The number of neoplasms per mouse was established by three independent researchers. (C) Average size of neoplasms per mouse in each group was measured as described in Materials and Methods. Only statistically significant differences are labeled (Tukey's post hoc test, *P *<* *0.05). C (control diet), CD (control diet with cyclodextrin), CP (control diet with propolis), W (Western diet), WD (Western diet with cyclodextrin), and WP (Western diet with propolis).

The average body weight gain in mice exposed to a control diet, control diet with gamma‐cyclodextrin, and control diet with propolis supplement, was comparable (5.8 ± 1.9, 5.9 ± 1.7, and 6.1 ± 0.9 grams, respectively, one‐way between groups ANOVA: *P *=* *0.960). The average body weight gain in mice exposed to a Western diet, Western diet with gamma‐cyclodextrin, and Western diet with propolis supplement, was 9.0 ± 4.3, 9.1 ± 2.0, and 5.2 ± 1.0 grams, respectively, one‐way between groups ANOVA: *P *=* *0.052 (Fig. 1A). Between all six intervention groups, one‐way ANOVA established significant differences in body weight gain [*F*(5, 26) = 3.2063, *P *=* *0.022]. Food consumption was similar between the mice on a control, control‐cyclodextrin, and control‐propolis diet (10.3, 8.8, and 8.3 kcal/mouse/day, respectively). Similarly, food consumption did not differ significantly between the mice on a Western diet, Western diet with gamma‐cyclodextrin, or Western diet with propolis (14.6, 13.0, 14.0 kcal/mouse/day, respectively).

### Neoplastic burden: number and size of intestinal neoplasms

Almost all neoplastic lesions were detected in the small intestine. There was a significant difference in the number of neoplasms between intervention groups on control diets as determined by one‐way ANOVA [*F*(2, 13) = 6.7687, *P *=* *0.0097], Figure 1B. The number of neoplasms in the group on a control diet without supplements was higher than in the group on a control diet with a propolis supplement (61.8 ± 10.6 vs. 35.3 ± 7.6, Tukey's post hoc test, *P *=* *0.008). There were no significant differences in the number of neoplasms between the mice on a control diet and the mice on a control diet with gamma‐cyclodextrin (43.6 ± 16.9), and between the two control diets with supplements (propolis or gamma‐cyclodextrin). Among the groups on Western diets, there were no significant differences in the number of neoplasms [*F*(2, 13) = 1.4521, *P *=* *0.270]; however, there were large fluctuations in the number of neoplasms among mice in the same intervention group; the mice on a Western diet without supplements had 47.6 ± 25.1 neoplasms, the mice on a Western diet with gamma‐cyclodextrin exhibited 27.8 ± 15.9 neoplasms, and the mice on a Western diet with a propolis supplement had 48.0 ± 23.0 neoplasms (Fig. 1B). Comparative analyses of all six interventions did not reveal statistically significant differences in the number of neoplasms [*F*(5, 26) = 2.2663, *P *=* *0.078], Fig. 1B.

Analyses of the mean size of the lesions were carried out between groups on control diets, groups on Western diets, as well as between all six dietary interventions (Fig. 1C). There were no significant differences in the neoplasm size between the three intervention groups on a control diet with or without supplements, as determined by one‐way ANOVA [*F*(2, 13) = 1.5166, *P *=* *0.256]. The mean size neoplasm in the group on a control diet without supplements was 5.64 ± 0.94 mm^2^, in the group on a control diet with gamma‐cyclodextrin ‐ 5.63 ± 0.90 mm^2^, and in the group on a control diet with a propolis supplement, 4.75 ± 1.07 mm^2^. There was a difference in neoplasm size between the three intervention groups on Western diets as determined by one‐way ANOVA [*F*(2, 13) = 18.1197, *P *=* *0.002]. A Tukey's post hoc test revealed that the neoplasm size in the group on a Western diet with gamma‐cyclodextrin (4.62 ± 0.82 mm^2^) was statistically significantly lower than the neoplasm size in the group on a Western diet without supplements (8.00 ± 0.68 mm^2^, *P *=* *0.001), and also than the neoplasm size in the group on a Western diet with a propolis supplement (6.33 ± 1.07 mm^2^, *P *=* *0.019). The neoplasm size in the group on a Western diet with a propolis supplement was also lower than that in the group on a Western diet without supplements (*P *=* *0.021). One‐way ANOVA that compared the effect of diet and supplements on the size of neoplasms in all six interventions revealed significant differences between the group means [F(5, 26) = 9.079, *P *=* *0.001].

When analyzing all six interventions, in addition to the above‐mentioned differences between each three treatments (in the context of control or Western diet), a Tukey's post hoc test to adjust for multiple comparisons with 95% confidence revealed differences (*P* < 0.01) between the mean values of neoplasm size in mice on all three control diets when compared to the neoplasm size in mice on a Western diet without supplements (Fig. 1C).

**Figure 2:**
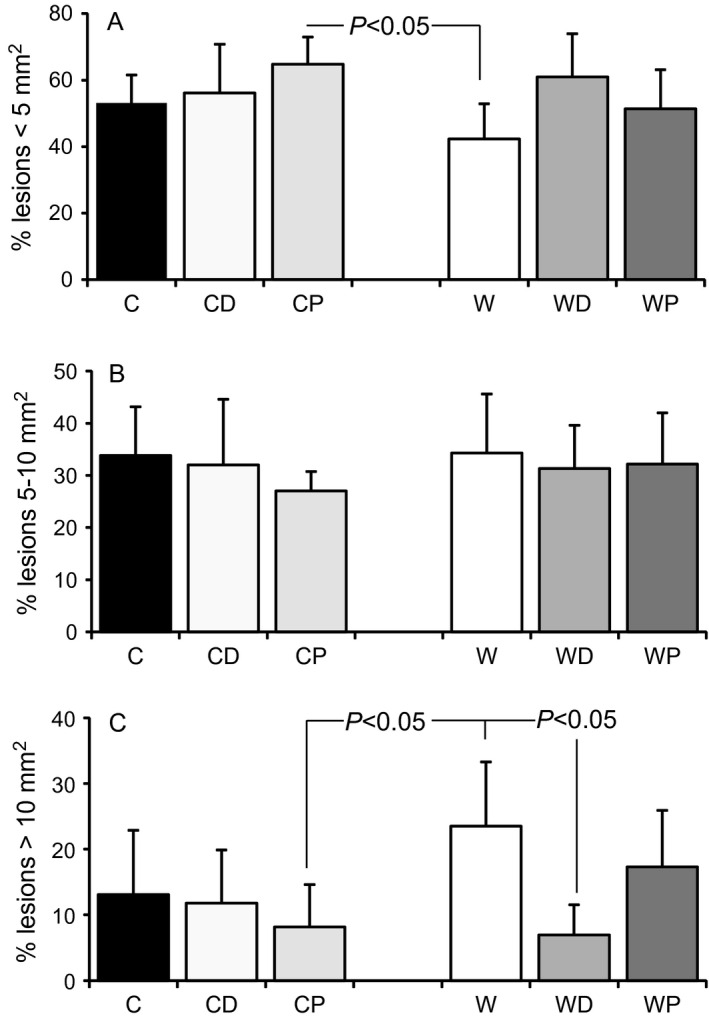
Number of neoplasms classified into three size categories: (A) small (<5 mm^2^), (B) medium (5–10 mm^2^), and (C) large (>10 mm^2^) in the six intervention groups: C (control diet), CD (control diet with cyclodextrin), CP (control diet with propolis), W (Western diet), WD (Western diet with cyclodextrin), and WP (Western diet with propolis). Only statistically significant differences are labeled (Tukey's post hoc test, *P *<* *0.05).

To ascertain differences in neoplastic progression among the six dietary intervention groups, we classified the neoplasms into three size categories: small (<5 mm^2^), medium (5–10 mm^2^), and large (>10 mm^2^). This categorization is justified by the range of average size lesions in all six intervention groups (the range was determined by the smallest size of 4.6 ± 0.8 mm^2^, detected in mice fed a Western diet with gamma‐cyclodextrin, and the largest size of 8.0 ± 0.7 mm^2^ in mice fed a Western diet). The percent neoplasms of medium size (5–10 mm^2^) did not differ in a statistically significant manner between the six dietary interventions [*F*(5, 26) = 0.4325, *P *=* *0.822] (Fig. 2B). However, the percent of small and large adenomas exhibited inverse correlation with the size of the lesions (Figs. 2A, C). The groups on supplemented control and Western diets exhibited higher numbers of small (<5 mm^2^, Fig. 2A) neoplasms and lower numbers of large (>10 mm^2^, Fig. 2C) neoplasms compared to the respective diets without supplements.

Analyses of the percent neoplasms with surface area greater than 10 mm^2^ (Fig. 2C) detected a significant difference among the six intervention groups [*F*(5, 26) = 3.0335, *P *=* *0.028]. A Tukey's post hoc test determined that the percent large (>10 mm^2^) neoplasm was higher in mice fed a Western diet without supplements (23.5 ± 9.8%) compared to the percent large neoplasms in mice fed a control diet with a propolis supplement (8.2 ± 6.5%, *P *=* *0.043) (W vs. CP, Fig. 2C). Also, the percent large (>10 mm^2^) neoplasm was higher in mice fed a Western diet without supplements (23.5 ± 9.8%) compared to that in mice fed a Western diet with gamma‐cyclodextrin (11.8 ± 8.1%, *P *=* *0.034) (W vs. WD, Fig. 2C). Analyses of the percent neoplasms smaller than 5 mm^2^ also detected a significant difference among the six intervention groups [*F*(5, 26) = 2.6228, *P *=* *0.048] (Fig. 2A). A Tukey's post hoc test revealed that the percent small (< 5 mm^2^) neoplasms was lower in mice fed a Western diet without supplements (42.3 ± 10.6%) compared to the percent small neoplasms in mice fed a control with a propolis supplement (64.8 ± 8.2%, *P *=* *0.030) (W vs. CP, Fig. 2A).

### Grade of the intestinal lesions

**Figure 3:**
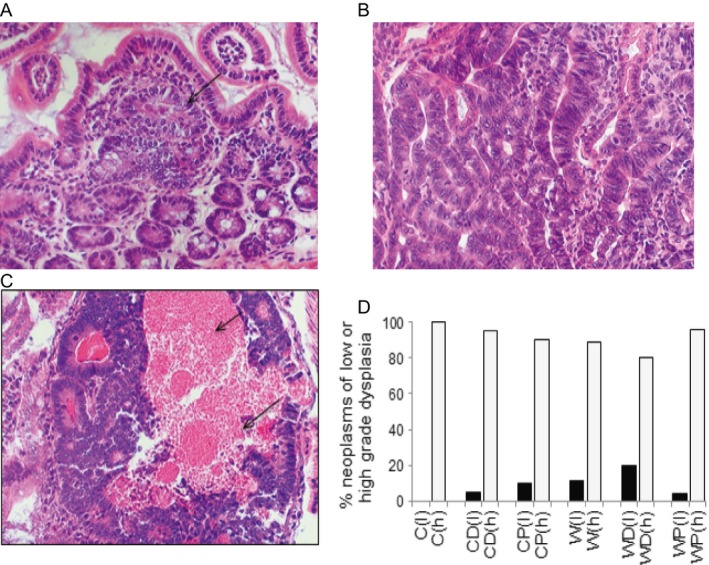
Histopathologic evaluation and grading of intestinal lesions detected in *ApcMin*/+ mice exposed to dietary interventions. (A) Mice on a Western diet with gamma‐cyclodextrin exhibited a higher number of adenomas of small size with less than six adenomatous glands. These glands look alike the surrounding normal ones with uniform nuclei with little dysplasia (A, arrow). (B) High‐grade dysplastic adenomas as the ones developed in mice on a Western diet displayed severe disorganization of the glandular architecture. Abnormal nuclear changes such as hyperchromatism, increase of the nuclear/cytoplasm ratio, irregular and pseudostratificated nuclei, conspicuous nucleoli, and mitosis were evident. (C) Adenomas developed in mice on a Western diet frequently displayed intraluminal necrosis (arrows, C). Histological sections stained with hematoxylin and eosin are shown (400 × original magnification). (D) Percent neoplasms with low (l) or high (h) grade of dysplasia were calculated based upon the analyses of the HE‐stained paraffin‐embedded mouse intestines within each intervention group (*n* = 5–6).

Our mouse model is limited to the precarcinoma stage. However, even at this stage, the size of adenomas strongly correlates with the grade dysplasia; large adenomas usually display high‐grade dysplasia, and *vice versa*, smaller in size adenomas exhibit lower grade of dysplasia **[** 26]. Since adding gamma‐cyclodextrin and propolis to control or Western diets decreased the number of large (>10 mm^2^) adenomas (Fig. 2C), we ascertained whether the lower number of large neoplasms corresponds to a lower grade of dysplasia. Low‐grade dysplasia was noted where branching/elongation of the crypts with reduced interglandular stroma occurred with low nuclear/cytoplasmic ratio and elongated and crowded cell nuclei (Fig. 3A). High‐grade dysplasia was characterized by high nuclear/cytoplasmic ratio, severe reduction of the interglandular stroma, cytologic and architectural changes, nuclei with loss of polarity with respect to the basal membrane, irregular nuclear membranes, and aberrant chromatin pattern (Fig. 3B). Among all intervention groups, the only signs of focal necrosis were observed in the neoplastic tissues of mice fed a Western diet without supplements (Fig. 3C). Among the control diets, in the group on a diet without supplements, all lesions were classified as high dysplasia, 5% were graded as low dysplasia in the control diet with gamma‐cyclodextrin, and 10% were graded as low dysplasia in the control diet with a propolis supplement (Fig. 3D). Among the Western diets, the mice on a diet with gamma‐cyclodextrin exhibited 20% of low grade lesions; whereas, the mice on a Western diet alone and a Western diet with propolis exhibited 11% and 4% low‐grade neoplasms, respectively (Fig. 3D).

### Molecular studies

**Figure 4:**
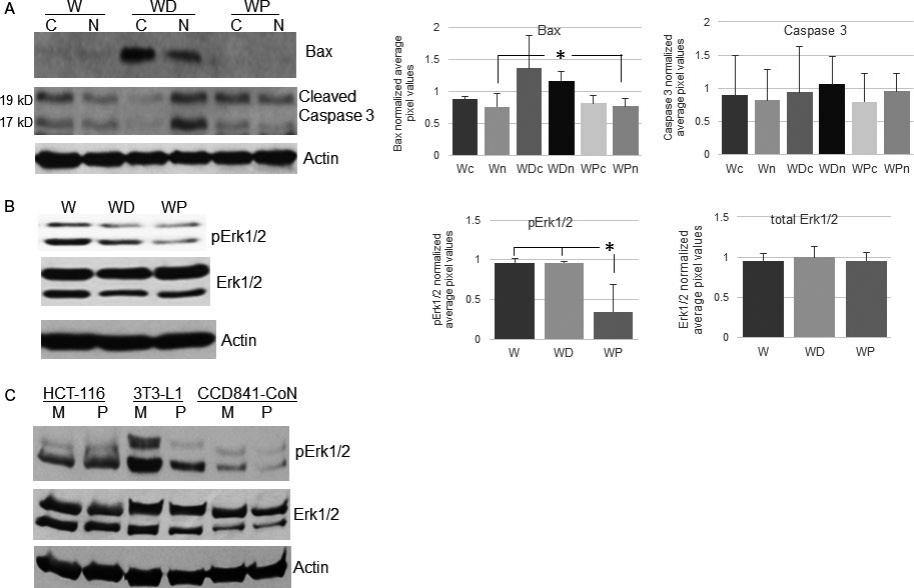
A. A representative western blot analysis of Bax and cleaved Caspase 3 in “normal” (C) with no discernible lesions tissues, and neoplastic (N) tissues of mice on a Western diet without supplements (W) or supplemented with gamma‐cyclodextrin (WD) or propolis (WP). B. A representative western blot analysis of ERK/pERK levels in the epididymal tissues of mice exposed to the three Western diets (W‐Western, WD‐Western and gamma‐cyclodextrin, WP‐Western and propolis). C. A representative western blot analysis of Erk/pErk levels in four cell lines exposed to mock treatment (M) or 100 *μ*g/mL propolis (P) for 16 h. Actin is a loading control. Densitometry was performed for four sets of sample analyses for Caspase 3 and three sets for each Bax, pErk1/2, and total Erk1/2. Asterisks designate the statistically significant differences in Actin‐normalized background‐corrected average pixel values.

Among the three intervention groups on a Western diet, the size of neoplasms was statistically different in the mice on a Western diet supplemented with gamma‐cyclodextrin. We ascertained whether this effect is due to the ability of gamma‐cyclodextrin to modify survival pathways in the intestinal neoplastic tissues. Western blot analyses with normal and neoplastic tissue lysates did not reveal consistent changes in Akt/pAkt, Stat3/pStat3, Erk/pErk, and c‐Jun/pc‐Jun levels (data not shown). Next, we tested whether the smaller size and lower number of neoplasms in mice fed a Western diet with gamma‐cyclodextrin are due to higher apoptosis in the intestinal tissues. The levels of the proapoptotic protein Bax and the cleaved Caspase 3 (a hallmark of apoptosis) were increased in the tissues of mice on a Western diet with gamma‐cyclodextrin (Fig. 4A).

Another observation of our *in vivo* analyses was that mice on a Western diet with propolis did not gain body weight to the same extent as mice fed a Western diet or a Western diet with gamma‐cyclodextrin (Fig. 1A). To explain this observation at the molecular level, we analyzed the major obesity‐related signaling pathways in the epididymal white fat tissues of mice fed Western diets with or without supplements. Western blot analyses revealed lower pErk1/2 levels in the epididymal white fat tissues of mice fed a Western diet with propolis (Fig. 4B); however, there were no consistent changes in the levels of Akt/pAkt, Stat3/pStat3, and pc‐Jun/c‐Jun (data not shown). We have previously reported that propolis hyperactivates ERK signaling in CC cells *in vitro* 16. To address this discrepancy, we analyzed the response to propolis in two normal cell lines: murine fibroblast 3T3‐L1 and human epithelial intestinal CCD841CoN. As we have reported, propolis augmented the levels of pERK1/2 in colorectal carcinoma HCT‐116 cells; however, pERK1/2 levels were decreased in the normal cells (Fig. 4C).

### Mutational profile of the neoplastic burden in mice on a control versus Western diet

We have reported that compared to normal weight patients, cancer genomes of obese microsatellite stable CC patients exhibit lower numbers of mutations in driver genes 4. To test whether this trend is observed in a murine model of intestinal cancer, we undertook exome sequencing of obese (Western diet‐fed) versus normal weight (control diet‐fed) mice (Fig. 1A). The analysis confirmed the germline *Apc* mutation in nine out of the 10 analyzed *Apc Miin/+* mice. The inability to detect the *Apc* germline mutation in one of the samples was due to low data size based upon low quality of the sample derived from a paraffin‐embedded intestine. We analyzed only mutations defined as moderate or high impact. High‐impact variants have disruptive impact in the protein (e.g., truncations, loss of function, causing nonsense mediated decay); whereas, moderate impact variants are nondisruptive variants that might change protein effectiveness (e.g., missense variants, in‐frame deletions, etc.). We did not include low‐impact variants in our analyses, since these mutations are mostly harmless or unlikely to change protein behavior (e.g., synonymous variants). Since the sequencing was performed on genomic DNA extracted from whole small intestines, we could not determine how many and what type of genes were affected in a single neoplastic lesion. All mice had multiple neoplasms; therefore, the number and type of affected genes refer to the sum of neoplastic lesions in each mouse. There was a high variation in the affected gene types between normal weight and obese mice; including *Apc* mutations, mice on a control diet exhibited moderate or high‐impact mutations in 51 types of genes; whereas, obese mice exhibited such mutations in 36 types of genes. Seven affected genes were common for the two groups of mice, in addition to *Apc*, these mutations affected *Akap*9, *Bcl*3, *Cdh*11, *Hsp90ab*1, *Hoxa*13, and *Nsd*1. There was also a great variation in the types of genes affected within each intervention group. In the control diet group, in addition to the *Apc* mutations, only mutations in *Akap*9, *Cdh*11, *Hoxa*13, and *Hsp90ab*1 occurred in more than one mouse. Among the obese mice, in addition to the *Apc* mutations, only mutations in *Akap*9, *Cdh*11, and *Hsp90ab*1 occurred in more than one mouse. In mice on a control diet, on average 12.2 ± 3.0 genes were affected by moderate‐ and high‐impact mutations; in obese mice, 10.4 ± 3.6 genes were affected by such mutations. The number of mutations per mouse was more than the number of affected genes, since there were several mutations detected in some genes. Although the difference was not statistically significant, normal weight mice exhibited more mutations than obese mice (30.6 ± 6.6 vs. 23.2 ± 9.0, respectively, *P *=* *0.177, Student's t‐test).

## Discussion

Obesity is a growing health care problem, as it is a risk factor for 10 types of cancer 5, 27, including CC; however, the mechanisms for this association are still unclear 4, 28. This study is the first translational step that follows upon our *in vitro* findings on how propolis modulates CC cells 14, 16, and it was designed to determine whether propolis influences intestinal neoplastic development *in vivo*, in the context of normal weight and obesity.

Among the groups of mice on control diets, the groups exposed to supplements, gamma‐cyclodextrin and propolis, decreased neoplastic progression, as determined by the mean size and number of all lesions, as well as by the grade of dysplasia. A statistically significant decrease in the number of adenomas was detected in mice fed a control diet supplemented with propolis, compared to mice fed a control diet without supplements. Compared to the mice on a control diet without supplements, the mice fed a control diet with gamma‐cyclodextrin also exhibited a decrease in lesion number and in grade of dysplasia; however, these differences were not statistically significant.

Among the three intervention groups on a Western diet, the differences in the number of neoplasms were not statistically significant; whereas, the differences in neoplasm size were statistically significant. The group with the lowest number/size of adenomas and the highest percentage of lesions with low grade of dysplasia was the group on a Western diet supplemented with gamma‐cyclodextrin. The group on a Western diet with propolis also exhibited a statistically significant decrease in the size of neoplasms when compared to the group of mice on a Western‐type diet without supplements.

Analyses of the neoplasm size among the six intervention groups revealed that the mice fed a Western diet without supplements exhibited statistically larger size neoplasms when compared to any other dietary intervention. This result confirms previous reports that a Western diet accelerates neoplastic progression 22, 29. However, unlike previous reports 23, under our experimental conditions, the mice on a Western diet did not exhibit statistically higher number of neoplastic lesions compared to the mice fed a control‐type diet. When the lesions were stratified in three categories by size, it became apparent that the mice on a Western diet had developed the highest number of large size neoplasms. The same analysis of neoplastic lesions stratified by size revealed a common trend among the six intervention groups. Thus, the intervention groups on supplements exhibited higher numbers of small neoplasms (<5 mm^2^) and lower numbers of large neoplasms (>10 mm^2^) in the context of the control or Western diet. There were no statistically significant differences among the numbers of medium size adenomas. A possible interpretation is that both supplements slowed the microadenoma to adenoma progression. However, whereas the propolis supplement was more potent in the context of a control diet, in the context of a Western diet, gamma‐cyclodextrin was more potent in reducing the neoplastic burden. Specifically, in obese state, compared to the Western diet alone, the supplementation with gamma‐cyclodextrin decreased both the size and number of lesions in a statistically significant manner, and the addition of propolis counteracted these effects. The lack of statistically significant difference between the numbers of neoplasms in mice fed a control diet and those fed a Western diet may arise from the fact in *ApcMin/+*/J mice, each somatic cell already carries a gatekeeping *Apc* mutation; therefore, we cannot determine the full impact of obesity on neoplastic initiation rate.

The histopathology evaluation of the neoplasms revealed that the larger size lesions in mice fed a Western diet correlated with faster progression; only the neoplasms in these mice exhibited focal necrosis. On the other hand, the lowest rate of neoplastic progression in mice fed a Western diet supplemented with gamma‐cyclodextrin was evidenced not only by the lowest number and the smallest size of neoplastic lesions but also by the histological evaluation of the tissues. This finding concurs with the observation of increased levels of apoptotic markers in the intestinal tissues of mice fed a Western diet with gamma‐cyclodextrin. In the intestines, gamma‐cyclodextrin could be fermented to short‐chain fatty acids, including butyrate 30, and when applied *in vitro*, butyrate induces apoptosis in cells with WNT/beta‐catenin signaling‐activating mutations, such as the mutations in *Apc* gene 10. Therefore, the increased apoptosis in the intestinal tissues of mice on a cyclodextrin‐supplemented diet could be due to the apoptotic effects of butyrate.

Our analyses revealed that unlike mice fed a Western diet without supplements or a Western diet with gamma‐cyclodextrin, the mice on a propolis*‐*supplemented Western diet did not gain excessive body weight. A similar effect of propolis on body weight in *C57BL/6N* mice fed a high‐fat diet has been reported 31; the authors demonstrated that propolis downregulates the expression of genes associated with lipid metabolism. Our analyses of major signaling pathways in the epididymal white fat tissues revealed suppressed pErk1/2 levels in mice fed a Western diet with propolis. It is not clear, however, whether the cell signaling changes are a cause for the suppressed body weight gain, or a result of this suppression. The lack of excessive adipose tissue could decrease the levels of the signaling pathway since obesity‐associated cytokines support ERK activity 32. However, propolis may also directly suppress ERK activity, and therefore, inhibit the formation of adipose tissues. For example, the levels of ERK signaling have a profound effect on adipocyte development 33, loss of ERK signaling inhibition leads to obesity in mice 34, and *erk*1‐/‐ mice resist obesity induced by high‐fat diet due to decreased adipogenesis and increased postprandial energy expenditure 33. We ascertained that the effect of propolis on ERK activity differs among normal and neoplastic cells; however, future studies are required to understand why propolis hyperactivates ERK signaling in neoplastic cells with *APC* and *KRAS* mutations (e.g., HCT‐116 cells) and suppresses the same pathway in normal cells (CCD841CoN and 3T3‐L1 cells) and adipose tissues of mice fed a high‐fat diet.

The faster neoplastic progression in obese mice fed a Western diet could be due to a higher mutation rate, increased number of stem cells and stem cell divisions, increased levels of adipokines, epigenetic changes, and changes in the intestinal microbiota 35. Some of these possibilities were addressed through exome sequencing analyses of normal weight (control diet‐fed) and obese (Western diet‐fed) mice. Although the average size of the neoplastic lesions in obese mice was larger than this in normal weight mice, the number and type of genes affected by moderate‐ and high‐impact mutations were lower in obese mice. This result supports our findings that compared to normal weight patients, cancer genomes of obese CC patients exhibit fewer somatic mutations, and correspondingly lower numbers of mutations in driver genes 4. The sequencing analyses did not reveal consistent differences in the type of affected genes. This could be due to the fact that neoplastic development in our experiment was limited to precarcinoma stage; whereas, the human exome analyses were performed on stage III colon carcinoma data from TCGA. However, the presence of multiple mutations even at the early adenoma stage has already been confirmed in humans 36, 37.

Currently, gamma‐cyclodextrin is *generally recognized as safe* (GRAS) by the U.S. Food and Drug Administration. The substance is added at 1% to 20% to food items such as dry mixes for beverages, soups, dressings, gelatins, instant coffee, cereals, snacks, dairy products, spreads, and is used as a carrier for vitamins and dietary supplements. The average daily intake of gamma‐cyclodextrin from its uses in foods has been estimated at 4.1 g / person / day; whereas, the estimate at the 90th percentile consumption level is 9.0 g / person / day 38. Propolis supplements are also available on the market; however, the constituents of propolis vary widely depending upon the geographic location where the honeybee product is collected. The utilized by us *Propolis with CycloPower Powder*, a product that will be commercially available in the second half of 2016, is tested and certified for the levels of eight bioactive compounds including caffeic acid phenethyl ester, chrysin, galangin, and pinocembrin. Therefore, the transition to human clinical studies that explore the effects of these supplements on intestinal neoplastic burden is possible; however, prior to such studies, expanded studies with a larger number of mice per intervention group and in an additional mouse model of intestinal cancer are warranted.

## Conflict of interest

None declared.

## Supporting information

Table S1. The number of neoplasms per mouse is the average of three independent counts performed by three investigators blinded for the treatment.

Table S2. Exome sequencing and analysis.
